# A Multimodal Large Language Model as an End-to-End Classifier of Thyroid Nodule Malignancy Risk: Usability Study

**DOI:** 10.2196/70863

**Published:** 2025-08-19

**Authors:** Gerald Gui Ren Sng, Yi Xiang, Daniel Yan Zheng Lim, Joshua Yi Min Tung, Jen Hong Tan, Chiaw Ling Chng

**Affiliations:** 1 Department of Endocrinology, Singapore General Hospital, 20 College Road, Academia Level 3, Singapore, 169856, Singapore, 65 63214377; 2 Data Science and Artificial Intelligence Laboratory, Singapore General Hospital, Singapore, Singapore; 3 Office of Insights and Analytics, SingHealth, Singapore, Singapore; 4 Department of Gastroenterology, Singapore General Hospital, Singapore, Singapore; 5 Department of Urology, Singapore General Hospital, Singapore, Singapore

**Keywords:** thyroid nodules, artificial intelligence, large language models, multimodal, risk stratification systems

## Abstract

**Background:**

Thyroid nodules are common, with ultrasound imaging as the primary modality for their assessment. Risk stratification systems like the American College of Radiology Thyroid Imaging Reporting and Data System (ACR TI-RADS) have been developed but suffer from interobserver variability and low specificity. Artificial intelligence, particularly large language models (LLMs) with multimodal capabilities, presents opportunities for efficient end-to-end diagnostic processes. However, their clinical utility remains uncertain.

**Objective:**

This study evaluates the accuracy and consistency of multimodal LLMs for thyroid nodule risk stratification using the ACR TI-RADS system, examining the effects of model fine-tuning, image annotation, prompt engineering, and comparing open-source versus commercial models.

**Methods:**

In total, 3 multimodal vision-language models were evaluated: Microsoft’s open-source Large Language and Visual Assistant (LLaVA) model, its medically fine-tuned variant (Large Language and Vision Assistant for bioMedicine [LLaVA-Med]), and OpenAI’s commercial o3 model. A total of 192 thyroid nodules from publicly available ultrasound image datasets were assessed. Each model was evaluated using 2 prompts (basic and modified) and 2 image scenarios (unlabeled vs radiologist-annotated), yielding 6912 responses. Model outputs were compared with expert ratings for accuracy and consistency. Statistical comparisons included Chi-square tests, Mann-Whitney *U* tests, and Fleiss’ kappa for interrater reliability.

**Results:**

Overall, 88.4% (6110/6912) of responses were valid, with the o3 model producing the highest validity rate (2273/2304, 98.6%), followed by LLaVA (2108/2304, 91.5%) and LLaVA-Med (1729/2304, 75%; *P*<.001). The o3 model demonstrated the highest accuracy overall, achieving up to 57.3% accuracy in Thyroid Imaging Reporting and Data System (TI-RADS) classification, although still remaining suboptimal. Labeled images improved accuracy marginally in nodule margin assessment only when evaluating LLaVA models (407/768, 53% to 447/768, 58.2%; *P*=.04). Prompt engineering improved accuracy for composition (649/1,152, 56.3% vs 483/1152, 41.9%; *P*<.001), but significantly reduced accuracy for shape, margins, and overall classification. Consistency was the highest with the o3 model (up to 85.4%), but was comparable for LLaVA and significantly improved with image labeling and modified prompts across multiple TI-RADS categories (*P*<.001). Subgroup analysis for o3 alone showed prompt engineering did not affect accuracy significantly but markedly improved consistency across all TI-RADS categories (up to 97.1% for shape, *P*<.001). Interrater reliability was consistently poor across all combinations (Fleiss’ kappa<0.60).

**Conclusions:**

The study demonstrates the comparative advantages and limitations of multimodal LLMs for thyroid nodule risk stratification. While the commercial model (o3) consistently outperformed open-source models in accuracy and consistency, even the best-performing model outputs remained suboptimal for direct clinical deployment. Prompt engineering significantly enhanced output consistency, particularly in the commercial model. These findings underline the importance of strategic model optimization techniques and highlight areas requiring further development before multimodal LLMs can be reliably used in clinical thyroid imaging workflows.

## Introduction

Thyroid nodules are highly prevalent, with as many as 35% of individuals having thyroid nodules on imaging [1]. Ultrasound is the first-line and most accurate imaging modality to assess thyroid nodules. The high prevalence of predominantly benign thyroid nodules in the general population led to the development of ultrasound risk stratification systems [2-4]. The American College of Radiology Thyroid Imaging Reporting and Data System (ACR TI-RADS) [4] was established to determine which nodules should have fine-needle aspiration or ultrasound follow-up by using 5 ultrasound feature categories and the maximum size of the nodule to derive a Thyroid Imaging Reporting and Data System (TI-RADS) classification ranging from 1 (benign) to 5 (highly suspicious). This system requires the assessment of 5 categories, or descriptors, of ultrasound features: composition, echogenicity, shape, margin, and calcifications (echogenic foci). While the ACR TI-RADS aims to enable the objective assessment of malignancy risk, significant real-life interobserver variability due to a lack of agreement in assigning these features has been reported [5,6]. In addition, the ACR TI-RADS suffers from low diagnostic specificity [7].

Large language models (LLMs) are artificial intelligence (AI) models using deep learning and artificial neural networks to handle large amounts of data, offering high-level predictive performance for varied tasks, including knowledge synthesis and health outcome prediction [8]. They are pretrained on large sets of extant human-generated information to learn patterns and probabilities that allow the synthesis of de novo content.

LLMs have been shown to perform well in medical question-answering tasks [9]. Performance can be further improved by fine-tuning on specific medical datasets [10], producing models such as Google’s MedPaLM-2. To date, most research in this field has focused on question-answering problems [8], with use for clinical decision support being a more recent but equally promising area of study [11].

Beyond text-based tasks, there has been a growing interest in the use of vision-language multimodal LLMs, such as Microsoft’s Large Language and Visual Assistant (LLaVA), which combines a visual encoder with a general LLM to allow it to synthesize both image and text data [12]. Like text-based LLMs, they can be further fine-tuned with domain-specific knowledge. For instance, LLaVA has been fine-tuned with a large-scale biomedical figure-caption dataset to create the medicine domain–specific Large Language and Vision Assistant for bioMedicine (LLaVA-Med) model [13]. As opposed to purely text-based interpretation of human-generated ultrasound reports, in which the main time-consuming task is still the radiologist’s report, this can allow seamless or end-to-end image interpretation and classification tasks, offering both time and process improvements in image-based clinical diagnostic tasks.

Many commercial AI apps have been developed to perform image segmentation and risk stratification for thyroid nodules [14,15]. However, these typically use traditional machine-learning models, which require extensive training and testing, and are furthermore limited in scope to only a single clinical problem. The use of LLMs offers the possibility of an “out-of-the-box” solution with less development time and expense. One previous approach has combined a distinct visual encoder model developed using classical machine learning methods with a set of commercial LLMs with comparable performance to human evaluators [16]. However, this approach is computationally demanding and has the risk of information degradation between steps, affecting overall output quality.

More recently, Cabezas et al [17] performed the first study evaluating OpenAI’s GPT-4 and 4o in performing the same task, but reported suboptimal accuracy, particularly for higher-risk nodules. However, the authors did not attempt optimization of the performance of these models, nor assess the consistency of outputs. LLM output is known to be inherently stochastic, with variability between the outputs. Clearly, a model that produced varying outputs for a single scenario would not be suitable for reliable clinical use. Hence, assessment of consistency is equally important as accuracy in evaluating the performance of such models. Finally, the use of commercial LLMs (such as OpenAI) for these tasks can be hampered by concerns about cost, patient privacy, and data protection. Open-source LLMs can be housed within health care institutions themselves, run with existing compute, and may be an alternative to address these concerns [18]. Furthermore, they typically have much fewer parameters and are hence less computationally demanding to run.

Therefore, we sought to explore the following objectives. First, to determine the accuracy and consistency of an open-source end-to-end vision-language model in the assessment of thyroid nodules using a standardized risk assessment system (ACR TI-RADS). Second, to study whether fine-tuning on medical domain knowledge improved the performance of the vision-language model for this task. Third, to evaluate whether the vision-language models were able to produce similar output without explicit human-labeled image segmentation. Fourth, to examine the effect of prompt engineering—a simple but effective method for improving the quality of LLM responses [19]—on the output of the model. Finally, we sought to evaluate the performance of this open-source model against a commercial LLM.

## Methods

### Materials

In total, 3 different models were evaluated in this study. First, Microsoft’s LLaVA model—which, while being putatively less powerful than other commercial multimodal LLMs [20], has the advantage of being open-source and can be easily instruction-tuned in a resource-efficient manner for medical domain knowledge [13]. Instruction tuning is a method of supervised fine-tuning in which models are trained on extant input-output pairs, as opposed to classical fine-tuning, which typically involves training a base model on a dataset of domain-specific parameters [21]. This instruction-tuned model, known as LLaVA-Med, was used as the second model for the study. The details of the instruction-tuning for LLaVA-Med have been described in the original article by Li et al [13]. Finally, the final model was the commercial OpenAI o3, which has been described by the company as their “most powerful reasoning model” in production [22].

Ultrasound cine-clip images, radiologist-annotated segmentations, and ACR TI-RADS descriptors for 192 different thyroid nodules from 167 patients were obtained from the publicly available dataset published by Yamashita et al [23]. This dataset is comprised of 175 benign and 17 malignant nodules, with an overall mean nodule size of 2.5 (SD 1.4) cm. The nodule characteristics, including breakdown of ACR TI-RADS level in each group, and image acquisition techniques have been described in detail in the original article.

**Figure 1.**
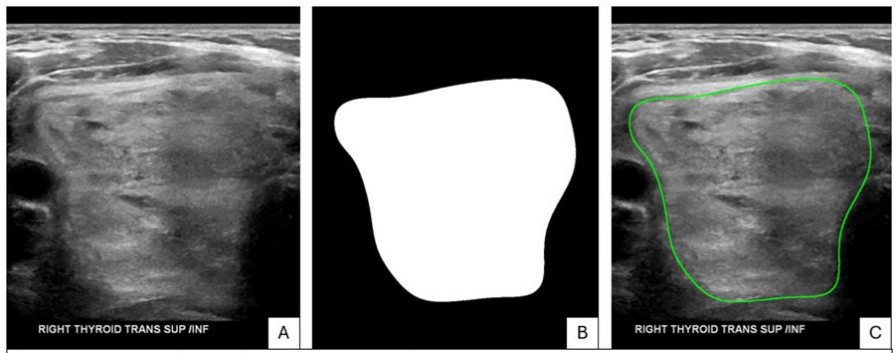
(A) Original unlabeled thyroid nodule ultrasound image. (B) Radiologist-annotated region of interest. (C) Composite overlay forming the submitted “labeled” image [23].

All models were queried with a prompt and individual ultrasound cine-clip images using an action-programming interface. The models were provided with unlabeled images and images labeled with radiologist-annotated regions of interest (ROI) as separate scenarios (Figure 1), for a total of 384 scenarios. As the models are only able to parse static images, only static images were provided, and the full cine-clips of each nodule were not used for this study.

### Data Collection

The first evaluation used a simple standardized prompt (“Basic Prompt”) detailing the scenario, task, and a summary of the ACR TI-RADS components. To improve the performance and reliability of the output, we repeated the evaluation after prompt engineering (“Modified Prompt”) with the following strategies. First, by contextualizing the task by including the descriptors of each component of the ACR TI-RADS, associated component point scores, and the overall point scores associated with each TI-RADS classification level. Second, by using task decomposition to break down the task into a series of ordered steps, in this case, by instructing the models to score each individual component, add up the sum of components, and finally translate the overall score to a TI-RADS classification level. Third, by constraining the model output into a specific format to minimize the rate of invalid responses. Both the basic and modified prompts are illustrated in Table 1.

**Table 1.** Basic and modified prompts.

Prompt	Text
Basic Prompt	Assume you are an assistant to a radiologist. This is an ultrasound image of the thyroid. The radiologist would like to know the points for each of the 5 components in Thyroid Imaging Reporting and Data System (TI-RADS) criteria for this image. Here are the 5 components: Composition. The point value ranges from 0 to 2 Echogenicity. The point value ranges from 0 to 3 Shape. It has only two point values: 0 or 3 Margin. The point value ranges from 0 to 3 Echogenic Foci. The point value ranges from 0 to 3
Modified Prompt	I would like you to assume the role of a radiologist’s assistant. This is an ultrasound image of a thyroid nodule. The radiologist would like to know the points of each of the 5 components of the Thyroid Imaging Reporting and Data System (TI-RADS), the total TI-RADS score, and the TI-RADS classification for this image. The 5 components and their points are as follows: Composition (choose one): Cystic or completely cystic: 0 Spongiform: 0 Mixed cystic and solid: 1 Solid or almost completely solid: 2 Echogenicity (choose one): Anechoic: 0 Hyper- or isoechoic: 1 Hypoechoic: 2 Very hypoechoic: 3 Shape (choose one): Wider than tall: 0 Taller than wide: 3 Margin (choose one): Smooth: 0 Ill-defined: 0 Lobulated/irregular: 2 Extra-thyroidal extension: 3 Echogenic foci (choose one or more): None: 0 Large comet-tail artifact: 0 Macrocalcifications: 1 Peripheral/rim calcifications: 2 Punctate echogenic foci: 3 Add up the points of each individual component to give the total points. Using the total points, the TI-RADS classification is as follows: 0 points: TI-RADS 1 2 points: TI-RADS 2 3 points: TI-RADS 3 4 to 6 points: TI-RADS 4 7 points or more: TI-RADS 5 Provide your output in the following format: Composition: Single integer Echogenicity: Single integer Shape: Single integer Margin: Single integer Echogenic foci: Single integer Total points: Single integer TI-RADS classification: String

**Figure 2.**
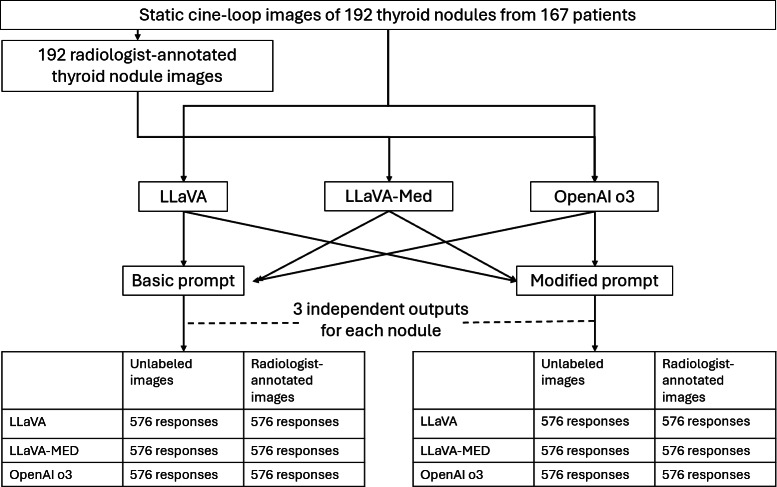
Schematic diagram of study design. LLaVA: Large Language and Visual Assistant; LLaVA-Med: Large Language and Vision Assistant for bioMedicine.

A schematic of the study design is shown in Figure 2. Using each prompt, 3 sets of responses from each model were obtained for each of the 384 scenarios, for a total of 6912 responses. Each response generated by the LLM consisted of 6 components—the 5 individual component scores (composition, echogenicity, shape, margin, and echogenic foci) and the overall TI-RADS classification level. However, as LLMs are known to be poor at basic arithmetic tasks [24], the study team separately added up the individual component scores manually to derive the overall TI-RADS classification level. While the modified prompt generated an additional seventh item – the sum of the individual component scores – this was designed purely as an intermediary step and was not included in the evaluation. Discrepancies between the LLM-generated and manually-calculated TI-RADS classification level were resolved by selecting the manually-calculated value for the analysis.

Evaluation using just the overall TI-RADS classification level alone may not be sufficiently robust, as nodules with different imaging characteristics may still derive the same overall classification. Take, for example, 2 nodules, both of which are solid, have regular margins, and are hypoechogenic. The first nodule may be taller than -wide (3 points) without echogenic foci (0 points), while the second nodule may be wider than tall (0 points) with punctate echogenic foci (3 points). However, both would still arrive at the same TI-RADS classification level. Therefore, each item in the response was analyzed individually for accuracy and consistency to demonstrate the models’ performance in evaluating each component of the TI-RADS classification, in addition to the overall TI-RADS classification level.

### Statistical Analysis

Accuracy of output was defined by comparison with the human-rated TI-RADS classification level obtained from the original dataset. Considering the intrinsic variability of human raters [5,6], the aggregate output was deemed to be “accurate” if at least 1 of the 3 sets of output was concordant with the human-rated score. This “best-of-three” approach has been proposed as a strategy to mitigate LLM output variability [25], and has been used in other studies evaluating the clinical performance of LLMs for a variety of medical tasks [26-29]. Model and human-rated median score distributions were compared in 2 ways. First, it was categorically based on whether the median score underestimated, equaled, or overestimated the TI-RADS classification level. Second, as the score distributions of both model and human-rated scores were determined to be nonparametric using the one-sample Kolmogorov-Smirnov test, they were therefore compared continuously using the Mann-Whitney *U* Test. Consistency of the output was assessed using 2 measures. First, the output was deemed “consistent” if all 3 sets produced the same result. Second, interrater reliability was compared using Fleiss’ kappa.

Differences in these metrics were analyzed by groups, namely, across all 3 models, unlabeled versus labeled scenarios, and basic prompt versus modified prompt. Differences in proportions were compared using the chi-square test.

Due to inherent model stochasticity, the models occasionally produced inappropriate or uninterpretable responses. Responses with any missing components, or where the output was a noninteger value (for instance, text or letters), were deemed invalid. As the purpose of this study was to evaluate the real-world model output, these invalid responses were still included in the final analyses for accuracy and consistency but were uniformly treated as inaccurate responses. Similarly, missing and noninteger values were still included in the calculation of interrater reliability.

Statistical analysis was conducted using the Python 3.10 environment, with the pandas 2.2.0 package for table processing, scipy 1.13.1 package for statistical analysis, and seaborn 0.13.2 for data visualization.

### Ethical Considerations

As the data used in this study were completely deidentified and freely available in the public domain, no ethical approval was required for this study.

## Results

### Overview

Out of the 6912 generated responses, 6110 (88.4%) were deemed valid. Use of the modified prompt improved the frequency of valid responses slightly from 87.6% to 89.2% (*P*=.04). The OpenAI o3 model produced the highest proportion of valid responses (2273/2304, 98.6%), followed by the LLaVA model (2108/2304, 91.5%), then the LLaVA-Med model (1729/2304, 75%, *P*<.001 for overall comparison). Most invalid responses were due to the inability of the LLM to interpret the provided image (“I am unable to view the image itself”). Some invalid responses were also due to the output of noninteger values (for instance, letter values ranging from a-e instead), or hallucinations that caused it to deviate from the assigned task (“In this particular image, the Ultrasound Institute of New York grading scale is used to assess the thyroid nodule”).

### Accuracy

**Figure 3.**
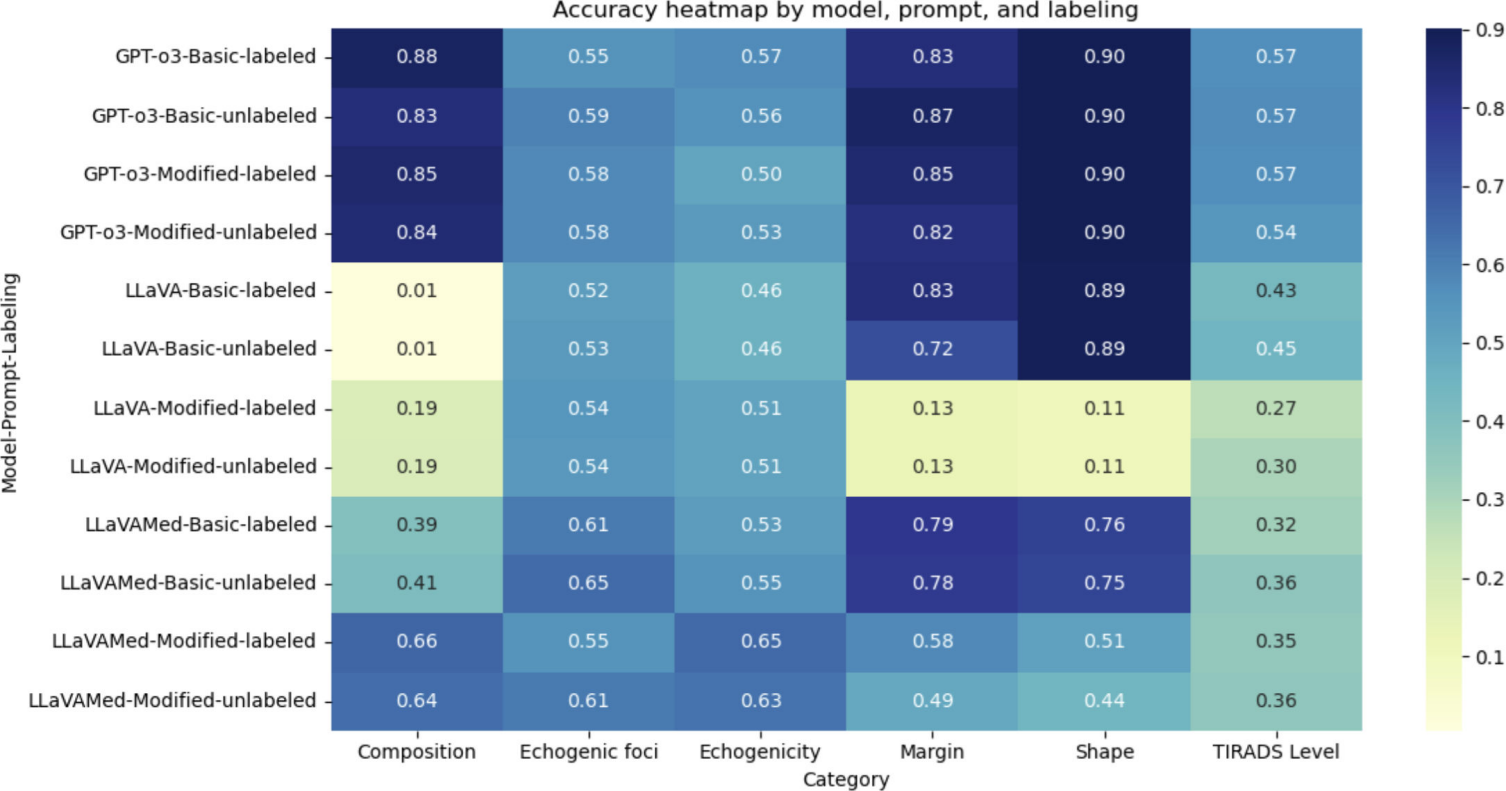
Heatmap showing the distribution of accuracy scores across all combinations of model, prompt, and labeling. LLaVA: Large Language and Visual Assistant.

The effect of the various combinations of model, type of prompt, and type of image on the accuracy of output for each component and the overall TI-RADS classification level is visualized as a heatmap in Figure 3. In general, the o3 model had the highest accuracy for most categories except echogenicity and echogenic foci (Table 2). LLaVA-Med appeared to be more accurate than o3 for these 2 categories, although the difference between the 2 models was not significant when compared pairwise (59% vs 54%, *P*=.06 for echogenicity; 60.7% vs 57.6%, *P*=.23 for echogenic foci). The best accuracy obtained for overall TI-RADS classification level was still only 57.3%.

**Table 2.** Accuracy of aggregate model output comparing between – Large Language and Vision Assistant, Large Language and Vision Assistant for biomedicine, and o3 models, unlabeled versus labeled scenarios, and base versus modified prompts.

Variable	Model selection				Image annotation			Prompt engineering		
TI-RADS^a^ component	LLaVA^b^	LLaVA-Med^c^	GPT-o3	*P* value	Unlabeled	Labeled	*P* value	Basic	Modified	*P* value
Composition	0.10	0.53	0.85	<.001	0.49	0.50	.71	0.42	0.56	<.001
Echogenicity	0.48	0.59	0.54	<.001	0.54	0.54	.90	0.52	0.55	.12
Shape	0.50	0.62	0.90	<.001	0.67	0.68	.53	0.85	0.50	<.001
Margin	0.45	0.66	0.84	<.001	0.64	0.67	.12	0.80	0.50	<.001
Echogenic Foci	0.53	0.61	0.58	.01	0.58	0.56	.27	0.58	0.57	.67
Overall TI-RADS^a^ classification level	0.36	0.35	0.56	<.001	0.43	0.42	.47	0.45	0.40	.01

a TI-RADS: Thyroid Imaging Reporting and Data System.

b LLaVA: Large Language and Vision Assistant.

c LLaVA-Med: Large Language and Vision Assistant for bioMedicine.

There was no one best combination of input variables in determining overall accuracy, with different combinations yielding the highest accuracy scores for each component as well as the overall TI-RADS classification level. Interestingly, the basic LLaVA model without prompt engineering or nodule annotation achieved the closest overall accuracy to the worst-performing combination using o3 (44.8% v 54.2%, respectively), despite very poor accuracy for composition and echogenicity.

When combining the output of all 3 models, the use of labeled (radiologist-annotated) images instead of unlabeled images (Table 2) did not make a significant difference to the accuracy of all component scores, nor the overall TI-RADS classification. However, secondary analysis done with output from just the LLaVA and LLaVA-Med models showed that there was a significant improvement in accuracy for margin classification from 53% to 58.2% (*P*=.04).

Use of the modified prompt as compared with the basic prompt (Table 2) significantly improved accuracy in classifying nodule composition (56.3% vs 41.9%, *P*<.001). However, there was worse accuracy in classifying nodule shape (49.6% vs 84.9%, *P*<.001) and margins (0.5% vs 80.3%, *P*<.001). Accuracy for the overall TI-RADS classification level was also worse (45% vs 39.8%, *P*=.01) using the modified prompt. There was no significant difference in the accuracy of classifying echogenicity and echogenic foci using either prompt.

There were no significant differences in median score distributions between the model output and human raters. However, when evaluated categorically, all 3 models appeared to consistently overestimate the scores for echogenicity and underestimate echogenic foci (Multimedia Appendix 1).

### Consistency

**Figure 4.**
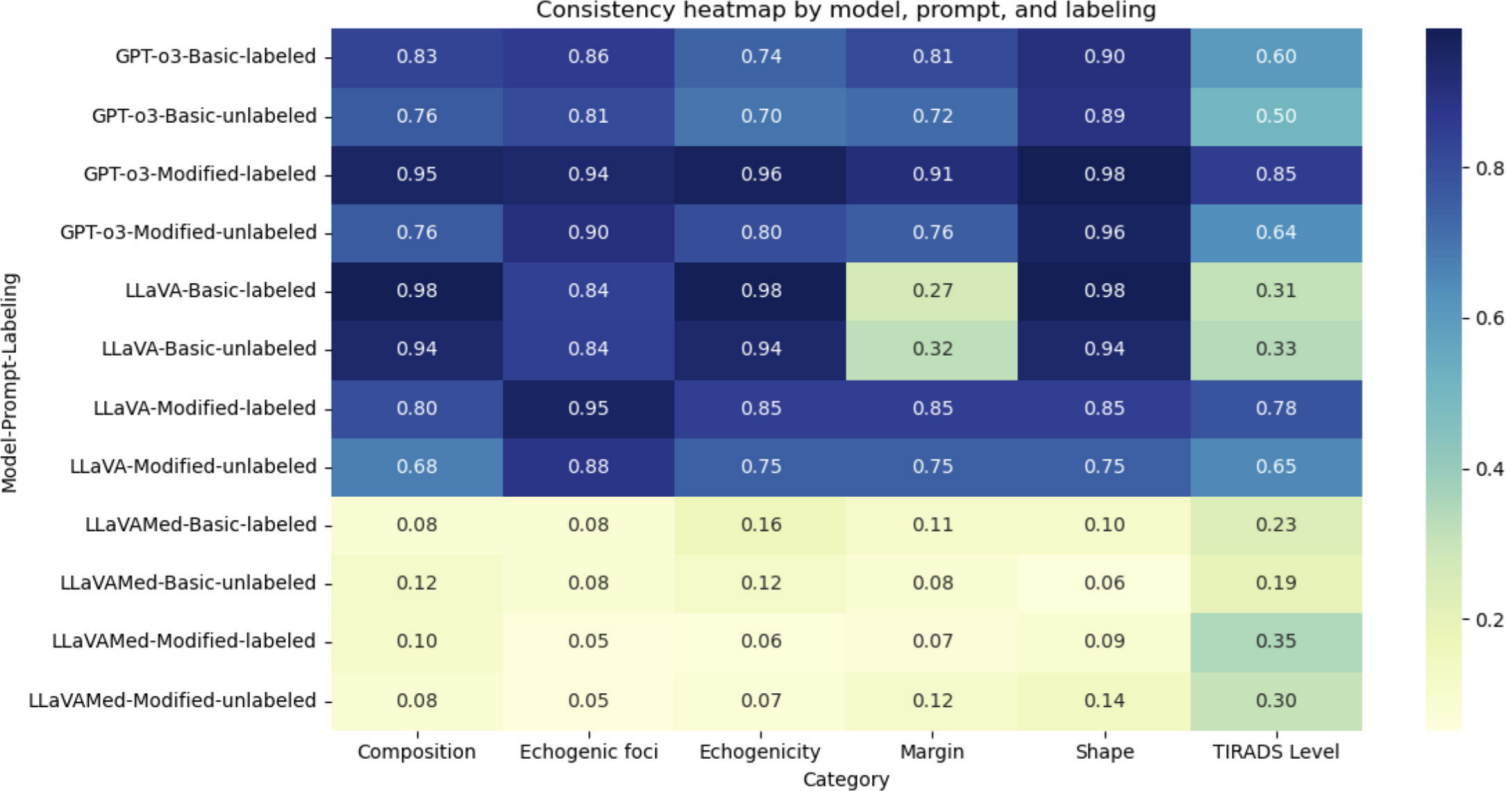
Heatmap showing the distribution of accuracy scores across all combinations of model, prompt, and labeling. LLaVA: Large Language and Visual Assistant.

The effect of the various combinations of model, type of prompt, and type of image on the consistency of output for each component and the overall TI-RADS classification level is visualized as a heatmap in Figure 4. The consistency of both o3 and LLaVA models was largely comparable across all categories except that of margins (80% vs 54.6% respectively, *P*<.001), while that of LLaVA-Med was significantly poorer than both across all categories (Table 3). The highest overall consistency of 85.4% was obtained from the combination of the o3 model with prompt engineering and annotated images.

**Table 3.** Consistency of aggregate model output comparing between – Large Language and Vision Assistant, Large Language and Vision Assistant for biomedicine, and o3 models, unlabeled versus labeled scenarios, and base versus modified prompts.

Variable	Model selection				Image annotation			Prompt engineering		
TI-RADS^a^ component	LLaVA^b^	LLaVA-Med^c^	GPT-o3	*P* value	Unlabeled	Labeled	*P* value	Basic	Modified	*P* value
Composition	0.85	0.10	0.82	<.001	0.56	0.62	.001	0.62	0.56	.008
Echogenicity	0.88	0.10	0.80	<.001	0.56	0.63	.003	0.61	0.58	.29
Shape	0.88	0.10	0.93	<.001	0.62	0.65	.14	0.65	0.63	.39
Margin	0.55	0.10	0.80	<.001	0.46	0.50	.03	0.39	0.58	<.001
Echogenic foci	0.88	0.07	0.88	<.001	0.60	0.62	.22	0.59	0.63	.04
Overall TI-RADS^a^ classification level	0.52	0.27	0.65	<.001	0.44	0.52	<.001	0.36	0.60	<.001

a TI-RADS: Thyroid Imaging Reporting and Data System.

b LLaVA: Large Language and Vision Assistant.

c LLaVA-Med: Large Language and Vision Assistant for bioMedicine.

When combining the output of all 3 models, use of labeled images instead of unlabeled images improved consistency of outputs for composition (62.3% vs 55.6%, *P*=.001), echogenicity (62.6% vs 56.3%, *P*=.003), margins (50.3% vs 45.7%, *P*=.03), and overall TI-RADS classification level (52% vs 43.8%, *P*<.001). There was no significant difference in consistency of outputs for shape and echogenic foci (Table 3).

Surprisingly, there was a mixed effect from use of the modified prompt as compared with the basic prompt (Table 3), with poorer consistency for composition (56.2% vs 61.7%, *P*=.008), but higher consistency for margins (57.5% vs 38.5%, *P*<.001), echogenic foci (62.9% vs 58.6%, *P*=.04), and overall TI-RADS classification level (59.5% vs 36%, *P*<.001). There was no significant difference between the type of prompt used for the consistency of rating echogenicity or shape.

Due to the relatively high frequency of invalid responses, interrater reliability was poor across all combinations of input variables, ranging between −0.15 and 0.49. No combination achieved a Fleiss’ Kappa above 0.60.

### Subgroup Analysis

As the o3 model appeared to perform the best for accuracy as well as consistency, subgroup analysis was performed on output from just the o3 model alone. Neither prompt engineering nor image labeling appeared to affect the accuracy of the output from o3 across all categories and for the overall TI-RADS classification level (Table 4).

**Table 4.** Accuracy and consistency of aggregate o3 model output comparing unlabeled versus labeled scenarios, and base versus modified prompts.

Metric	Accuracy						Consistency					
Variable	Image annotation			Prompt engineering			Image annotation			Prompt engineering		
TI-RADS^a^ component	Unlabeled	Labeled	*P* value	Basic	Modified	*P* value	Unlabeled	Labeled	*P* value	Basic	Modified	*P* value
Composition	0.84	0.87	.31	0.85	0.85	≥.99	0.76	0.89	<.001	0.79	0.85	.04
Echogenicity	0.54	0.54	.89	0.57	0.52	.19	0.75	0.85	.001	0.72	0.88	<.001
Shape	0.90	0.90	≥.99	0.90	0.90	≥.99	0.92	0.94	.39	0.90	0.97	<.001
Margin	0.85	0.84	.84	0.85	0.84	.69	0.74	0.86	<.001	0.55	0.75	<.001
Echogenic foci	0.59	0.57	.61	0.57	0.58	.94	0.86	0.90	.10	0.84	0.92	<.001
Overall TI-RADS^a^ classification level	0.56	0.53	.47	0.57	0.52	.15	0.57	0.73	<.001	0.55	0.75	<.001

a TI-RADS: Thyroid Imaging Reporting and Data System.

Significant differences were, however, still observed for consistency. Use of the modified prompt instead of the basic prompt improved consistency across all categories for echogenicity (85.4% vs 79.4%, *P*=.04), echogenicity (88.3% vs 71.9%, *P*<.001), shape (97.1% vs 89.6%, *P*<.001), margins (83.3% vs 76.6%, *P*=.02), echogenic foci (91.9% vs 83.6%, *P*<.001), and overall TI-RADS classification level (74.5% vs 54.9%, *P*<.001). Use of labeled images instead of unlabeled images improved consistency for composition (88.8% vs 76%, *P*<.001), echogenicity (85.2% vs 75%, *P*=.001), margins (86.2% vs 73.7%, *P*<.001), and overall TI-RADS classification level (72.7% vs 56.8%, *P*<.001), but did not make a difference for shape and echogenic foci. These findings are illustrated in Table 4.

## Discussion

### Principal Findings

To our knowledge, this is the first study to evaluate the use of an open-source multimodal vision-language model alongside a commercial multimodal LLM for the end-to-end risk stratification of thyroid nodules. Furthermore, this is also the first study to consider the important parameter of inter-output variability in assessing model performance for thyroid nodule assessment.

We believe our evaluation of both an open-source smaller model and a more powerful commercial model is important, as it provides a helpful guide for model selection for future development of clinically deployable apps. The putatively most powerful model may not always be the most suitable for a given task, due to reasons of cost, data privacy protection, or compute access. For instance, OpenAI o3 has a token cost of US $10 per 1 million input tokens and US $40 per 1 million output tokens. We consumed a total of 2.79 million input and 1.75 million output tokens to generate the 2304 outputs for this study, incurring a total cost of approximately US $98. By contrast, the LLaVA and LLaVA-Med models were run off a single GPU with no cost beyond the marginal cost of utilities. Furthermore, we were unable to test real-world clinical images on the commercial LLM in this study as our national data privacy regulations prevent us from sharing patient-derived data outside the secure health care computing environment, even if fully anonymized, whereas the LLaVA and LLaVA-Med models could potentially be run using existing hardware within our secure environment.

Use of an end-to-end vision-language model is likely to be more efficient and translatable to other clinical tasks as compared with traditional machine learning predictive models. However, while the newer, more powerful commercial model outperformed the older, smaller open-source model on most metrics of assessment, even the best model output was suboptimal despite optimization. We offer some suggestions for why this may be the case.

First, input of image data directly into a vision-language model can be technically challenging. Modern medical imaging is typically high-resolution and therefore feature-rich. However, current-generation multimodal LLMs struggle with visual identification when feature counts are high [30], with a consequent detrimental impact on performance. Even classical machine learning models perform optimally at lower image resolutions [31]. Conceptually, the assessment of thyroid nodule composition, echogenicity, and the presence of echogenic foci requires the ability to segment the nodule into multiple ROIs, and thereafter, differentiate between the characteristics of each ROI. For instance, to determine if a nodule is part-solid, a human evaluator would first distinguish that multiple areas of acoustic impedance exist within the nodule, then determine that some are more echogenic than others. Similarly, determination of hyper- or hypoechogenicity requires comparison with normal thyroid parenchyma. As this requires the processing of many features, it is perhaps unsurprising that model accuracy was generally poor for these components. Conversely, when evaluating shape or margins, the nodule can be treated as a whole ROI, which may have been why model accuracy was generally higher for these components. In addition, the different models in this study process images differently. LLaVA [12] and LLaVA-Med [13] tokenize images based on fixed token counts for image area before input into the LLM, whereas o3 can process images dynamically using a unified encoder [32]. This may explain in part the improved (but still suboptimal) accuracy of o3 for echogenicity and echogenic foci.

**Figure 5.**
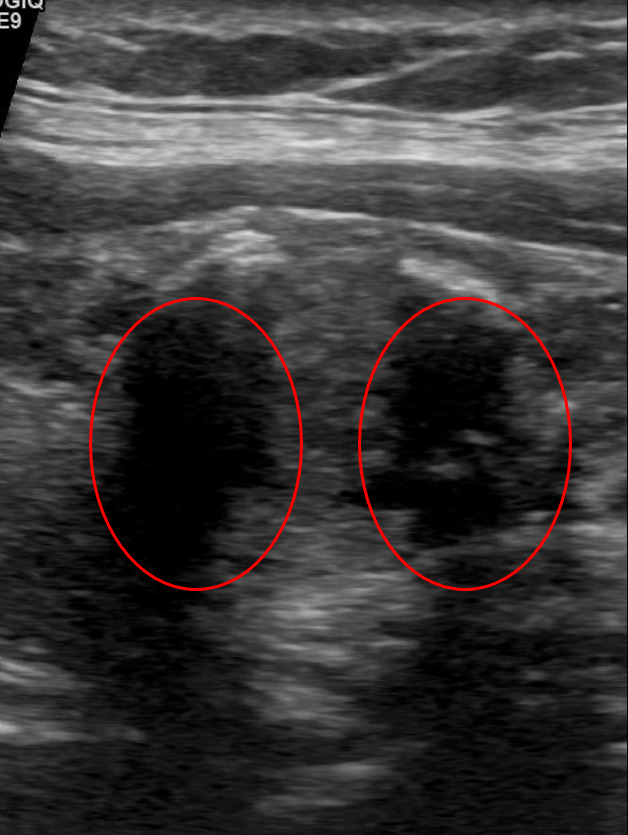
Ultrasound image from case 3 in the original dataset, showing a nodule with partial rim calcifications and consequent posterior acoustic shadowing (red circles). The nodule composition was erroneously classified as cystic (0 points) or mixed solid and cystic (1 point) in 19 out of 24 outputs, as opposed to almost completely solid (2 points) by the human rater.

**Figure 6.**
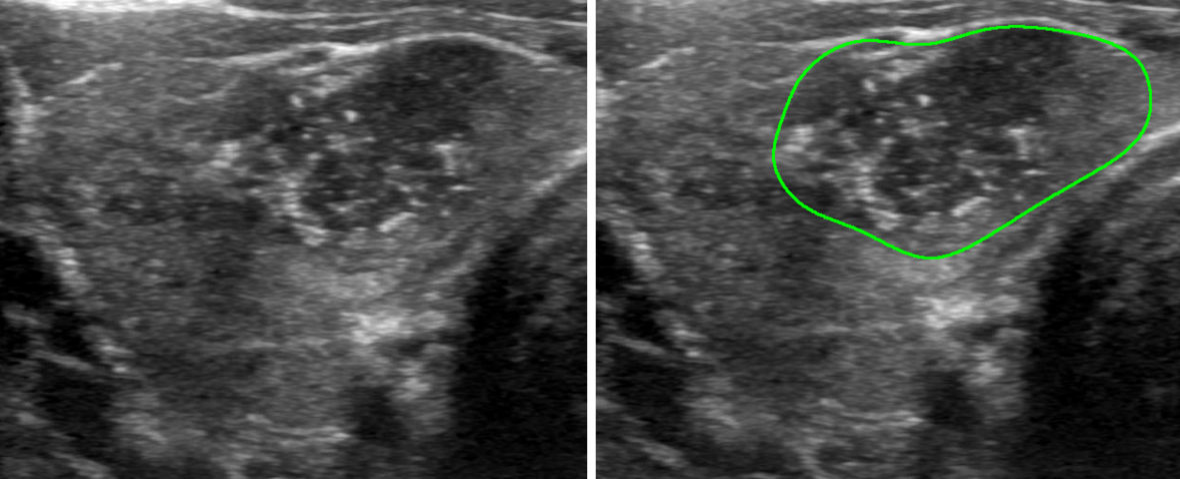
Unlabeled (left) and labeled (right) images from Case 132 in the original dataset. In total, 10 out of 12 outputs, using the unlabeled image, overestimated the nodule margins as irregular (2 points), while 6 out of 12 outputs using the labeled image overestimated the nodule margins as irregular (2 points). The human rater classified the nodule margin as ill-defined (0 points).

Second, vision-language models have reduced sensitivity to black-white contrast and have difficulty distinguishing between black and noise [30]. This is a limitation particularly relevant to ultrasound imaging, where regions that appear black can be anechoic or reflect the presence of acoustic shadowing. Distinguishing these can have a significant difference in the evaluation of thyroid nodules, as anechoic nodules are characterized as cystic and require no further evaluation, but acoustic shadowing typically signifies significant calcification, which is a risk feature for malignancy. An example of this is illustrated in Figure 5. In addition, nodule margins are typically recognized by differences in voxel density, which the models may struggle with if differences are small, and therefore use of images with human-annotated margins improved the classification of nodule margins for the LLaVA and LLaVA-Med models and consistency for all 3 models. An example of this is similarly demonstrated in Figure 6.

Intuitively, fine-tuning a general LLM with medical domain–specific knowledge should improve its performance for medical-specific tasks [33]. However, the nature of the data used for fine-tuning matters. LLaVA-Med did appear to have higher accuracy than LLaVA for all TI-RADS components evaluated in this study. Despite this, it was not more accurate than LLaVA in determining the overall TI-RADS classification level. To explain this, we noted that ultrasound images were not included in the LLaVA-Med fine-tuning figure-caption dataset. Furthermore, fine-tuning may have led to the poorer LLaVA-Med model consistency observed, as it would have been forced to sample from a probability distribution more skewed toward information irrelevant to its task and therefore produce a wider range of responses as a result.

Finally, prompt engineering significantly improved the consistency of output across all categories, including the overall TI-RADS classification level. This improvement was consistently observed regardless of the model used. This suggests that prompt engineering can still be a useful technique to optimize the output of “higher-performance” current-generation commercial models and should still be considered as a simple optimization technique in studies performed using these models.

However, prompt engineering had a mixed effect on accuracy. Furthermore, in the subgroup analysis on o3 output alone, there was no difference in accuracy, suggesting that the main effect of prompt engineering on accuracy was only seen in the open-source models. Prompt engineering in text-only LLMs can be highly effective as the text prompt is the only determinant of the output [34]. In this case, the main limiting step in accuracy was likely not the knowledge of the individual categories of the TI-RADS classification system, but the knowledge of what the categories meant. To illustrate this, a human trainee looking at a thyroid nodule image for the first time would not be expected to know the meaning of “hyperechogenic” or “hypoechogenic.” He or she could refer to sample images showing the differences between these and extrapolate these observations to classify other nodules. Similarly, it might be unrealistic to expect the base vision-language model to intrinsically understand and translate the meaning of these classifiers.

Conversely, as O3 has a parameter count that is likely a few orders of magnitude higher than that of the open-source model (exact count not disclosed by OpenAI), it is conceivable that this “knowledge” is part of its pretraining set, hence also contributing to the better performance. Expanding on that hypothesis, a possible approach to further improve the output of smaller models could be to use few-shot prompting to provide it with the requisite examples to assist in subsequent classification. However, there are 19 different classifiers across the 5 TI-RADS components, and providing 19 example images in every prompt is costly and inefficient. Further work is required to explore the development of more efficient techniques of few-shot prompting to improve model performance for visual classification tasks, particularly for complex risk scores like this one.

This study has some limitations. LLM output is stochastic, or random, due to the inherent randomness of sampling techniques (such as top-k or nucleus sampling) used to derive the output. Stochasticity of output can be controlled by setting the “temperature” of the model, which is a measure ranging from 0 to 1 with 0 representing the least stochastic and 1 representing the most stochastic output ranges. We did not vary the base temperature in this study, as part of the objective was to measure the intrinsic model consistency. Using a low-to-zero temperature in future approaches is likely to improve consistency and consequently reproducibility of results.

Furthermore, the models used were only able to parse static images and not cine-clip images. It is arguable that certain features, such as punctate echogenic foci, are better appreciated on cine-clip images. However, real-world radiologists still appear to perform more consistently on static images rather than cine-clip images [35]. Therefore, the extent to which this limitation affects performance is unclear and should be evaluated further in other studies.

Next, the use of AI in health care is desirable because it may help to improve automation and productivity. Human calculations were still required in some cases in this study, which may have inflated the performance of the LLM. Rather than relying on the LLM alone to perform calculations, providing simple arithmetic tools to the LLM in an agentic framework may be an alternative solution to overcome this limitation.

Furthermore, human labeling was still used as an optimization strategy in this study. However, recent studies have explored the combination of LLM-generated prompts with a vision foundation model to perform zero-shot image segmentation, such as in the text-visual-prompt segment anything model (TV-SAM) algorithm incorporating GPT-4, the grounded language-imaging pre-training model, and the segment anything vision language model [36]. Image segmentation using such an algorithm, followed by interpretation of the segmented image with a classical multimodal LLM, may be a promising way to deliver a truly integrated end-to-end automated workflow in clinical practice and merits further exploration.

In addition, the test set used for evaluation in this study was from a single open-source dataset, which had a class imbalance between benign and malignant nodules (175 vs. 17, respectively). While this approximates the real-life distribution of thyroid nodules, whereby the vast majority are benign, it is possible that model performance on malignant cases may have been underpowered. Exploring these factors is beyond the scope of this exploratory study. This should be evaluated further in follow-on studies, which can also explore the generalizability to other datasets.

Finally, explainability (or the lack thereof) is a frequent criticism of end-to-end machine learning solutions, particularly in medicine, where safety and generalizability are concerns [8]. One method to improve explainability is to ask the LLM for explanations of how its output was derived [37]. We did not address this in our study as it was felt to be a moot point since overall performance was suboptimal.

LLMs are unique AI models because they are generally trained and can be easily repurposed for a variety of similar tasks. The major limitation for use in ultrasound image classification at present is in image processing and encoding, which can potentially be improved by relevant fine-tuning and improving feature extraction. This offers an alternative to resource-intensive model-building or fine-tuning and potentially retains flexibility for application in other imaging classification tasks. By addressing these limitations, we believe that computer-vision–assisted LLMs may eventually have the potential to augment human vision for visual-based classification tasks in medicine.

### Conclusions

With the rapidly evolving nature of the field of AI and LLMs in health care, we believe that no one fixed model can be expected to balance all the competing demands of performance, cost, deployability, and security. The findings from this study highlight the potential benefits of simple processes, such as prompt engineering and basic image preprocessing, to model performance. We further demonstrate that some of these techniques remain applicable even with the release of new, more powerful models. Overall, we believe the findings from this study provide formative insights for developers and researchers for further work in this area.

## Supplementary material

10.2196/70863 Multimedia Appendix 1 Categorical comparison of median model-generated scores against human-rated scores for each of the TI-RADS components and the overall TI-RADS classification. (A) Output from LLaVA and LLaVA-Med. (B) Output from OpenAI o3. (C) Output from all three models combined.

## Abbreviations

ACR TI-RADS: American College of Radiology Thyroid Imaging Reporting and Data System
AI: artificial intelligence
LLaVA: Large Language and Visual Assistant
LLaVA-Med: Large Language and Vision Assistant for bioMedicine
LLM: large language model
ROI: regions of interest
TI-RADS: Thyroid Imaging Reporting and Data System
