# Supplementary figures and images for: A Multimodal Large Language Model as an End-to-End Classifier of Thyroid Nodule Malignancy Risk: Usability Study
Source: JMIR Form Res. 2025 Aug 19;9:e70863. doi: 10.2196/70863 (PMC12364431; doi:10.2196/70863)

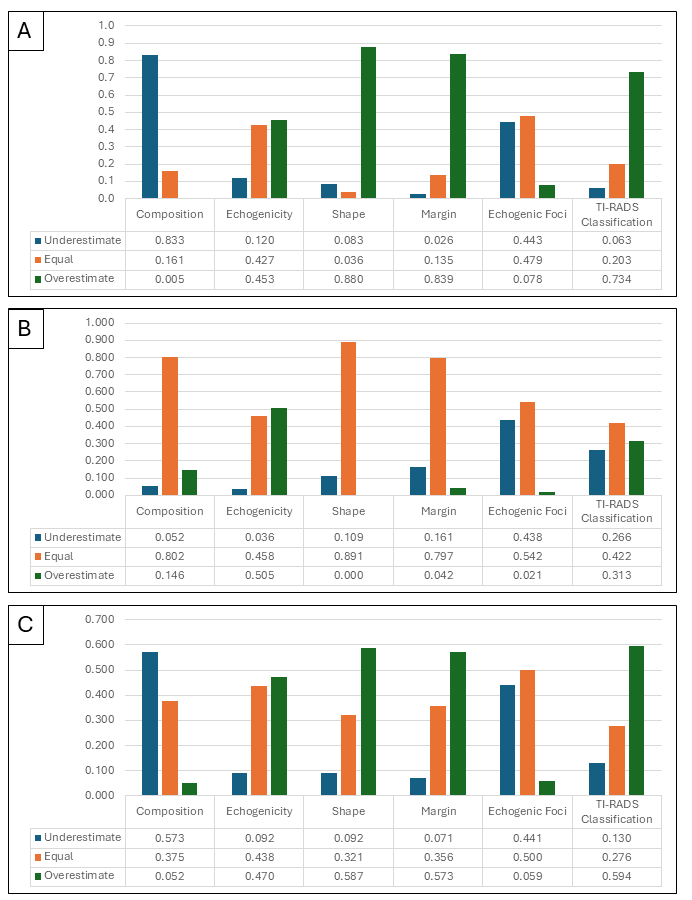

Supplement: Multimedia Appendix 1 [file formative-v9-e70863-s001.png]
